# New Approach toward Laser-Assisted Modification of Biocompatible Polymers Relevant to Neural Interfacing Technologies

**DOI:** 10.3390/polym13173004

**Published:** 2021-09-04

**Authors:** Nadya Stankova, Anastas Nikolov, Ekaterina Iordanova, Georgi Yankov, Nikolay Nedyalkov, Petar Atanasov, Dragomir Tatchev, Eugenia Valova, Konstantin Kolev, Stephan Armyanov, Daniela Karashanova, Naoki Fukata

**Affiliations:** 1 Institute of Electronics, Bulgarian Academy of Sciences, 72 Tzarigradsko Shousse Blvd., 1784 Sofia, Bulgaria; anastas_nikolov@abv.bg (A.N.); nnn_1900@yahoo.com (N.N.); paatanas@ie.bas.bg (P.A.); 2 Institute of Solid State Physics, Bulgarian Academy of Sciences, 72 Tzarigradsko Shousse Blvd., 1784 Sofia, Bulgaria; ekiordanova@gmail.com (E.I.); mennefer2@gmail.com (G.Y.); 3 Institute of Physical Chemistry, Bulgarian Academy of Sciences, Acad. Georgi Bonchev Str. Bld.11, 1113 Sofia, Bulgaria; dtachev@ipc.bas.bg (D.T.); jenny@ipc.bas.bg (E.V.); konstantinkolev07@gmail.com (K.K.); armyanov@ipc.bas.bg (S.A.); 4 Institute of Optical Materials and Technologies, Bulgarian Academy of Sciences, G. Bonchev Street, Bl. 109, 1113 Sofia, Bulgaria; dkarashanova@yahoo.com; 5 International Center for Materials for Nanoarchitectonics (MANA), National Institute for Materials Science (NIMS), 1-1Namiki, Tsikuba 305-0044, Japan; fukata.naoki@nims.go.jp

**Keywords:** ns-laser ablation in water, PDMS polymer, electroless metallization, platinum Pt black, neural interfacing technologies, implants

## Abstract

We report on a new approach toward a laser-assisted modification of biocompatible polydimethylsiloxane (PDMS) elastomers relevant to the fabrication of stretchable multielectrode arrays (MEAs) devices for neural interfacing technologies. These applications require high-density electrode packaging to provide a high-resolution integrating system for neural stimulation and/or recording. Medical grade PDMS elastomers are highly flexible with low Young’s modulus < 1 MPa, which are similar to soft tissue (nerve, brain, muscles) among the other known biopolymers, and can easily adjust to the soft tissue curvatures. This property ensures tight contact between the electrodes and tissue and promotes intensive development of PDMS-based MEAs interfacing devices in the basic neuroscience, neural prosthetics, and hybrid bionic systems, connecting the human nervous system with electronic or robotic prostheses for restoring and treating neurological diseases. By using the UV harmonics 266 and 355 nm of Nd:YAG laser medical grade PDMS elastomer is modified by ns-laser ablation in water. A new approach of processing is proposed to (i) activate the surface and to obtain tracks with (ii) symmetric U-shaped profiles and (iii) homogeneous microstructure This technology provides miniaturization of the device and successful functionalization by electroless metallization of the tracks with platinum (Pt) without preliminary sensitization by tin (Sn) and chemical activation by palladium (Pd). As a result, platinum black layers with a cauliflower-like structure with low values of sheet resistance between 1 and 8 Ω/sq are obtained.

## 1. Introduction

New materials and technologies have been developed in the last two decades for producing multielectrode arrays (MEAs) as interface networks of monitoring and/or stimulation of neural activity and connecting the human nervous system with electronic or robotic prostheses. R. Kim et al. [1,2] and Laing Guo et al. [3] have summarized in detail the fabrication steps of various types of planar multielectrode arrays in sandwich structures for in vitro and in vivo neural interface networks by reviewing technologies’ development. Briefly, the fabrication steps of such multielectrode structure are the following: choice of material like an insulator, to provide a good electrical signal with minimum attenuation and crosstalks; metal patterning, which includes an engineering design of the number and size of electrodes, interspace interval between electrodes, wire lines, electrical contact pads, metal conductor, and its electrical characteristics (impedance). Medical grade poly-dimethylsiloxane (PDMS) elastomer is a widely used material in medicine as a carrier material of shunts, pacemakers, and long-term neural implants [1,2,3,4,5,6,7,8,9]. In the case of the neural implant application, this synthetic polymer is highly preferred for insulator substrate and covering layer for performing stretchable MEAs neural interface devices [1,2,3,4,5,6,7,8,9,10,11]. This is the best alternative for the rigid insulator materials [1,2,3] (such as silicon dioxide, silicon nitride, or polymers (polyimide, parylene, acrylic imide) with a much higher Young’s modulus than of PDMS). The rigid MEAs carriers yield mechanical mismatch and can injure the soft bio-tissues. Medical grade PDMS elastomers can be synthesized as highly flexible with tunable mechanical properties due to the curing temperature and contents ratio. Samples with low Young’s modulus of about ~1.0 MPa can be obtained with values much closer to those of the nerve (600 kPa), muscles (usually <100 kPa) and brain (3 kPa) tissues [3] than the other insulator materials. Therefore, the PDMS-based MEAs neural devices can easily adjust to the soft tissue surface, conforming to its shape, and ensure tight contact between the electrodes and the neurons, thus optimizing the biological signal recording and/or stimulation. In addition, medical grade PDMS elastomers possess high cytocompatibility and biocompatibility, long-term implant life without disruption of the soft tissues (brain, muscles, and nerves), non-toxicity, high gas permeability, chemical stability, non-flammable, high dielectric breakdown, and high optical transparency in the UV-VIS-NIR spectral range. All these remarkable properties have promoted the intensive research and development of PDMS-based MEAs interface devices in basic neuroscience, neural prosthetics, and hybrid bionic systems, connecting the human nervous system with electronic or robotic prostheses [1,2,3,4,5,6,7,8,9,10,11]. Furthermore, all these hi-tech processes have been devoted to partial restoring of the motor and sensor functions (of limbs or organs) or the cognitive functions lost due to disease or trauma. Simply said, in a human aspect, these scientific efforts are being made for improving the life of people with disabilities. It is worth noting the development of advanced bioelectronic technology referred to as the “bionic eye”. This is an implanted retinal prosthesis consisting of various number electrodes (55–60 in case of platinum), packed in silicone (PDMS) insulator carrier, for stimulating retinal cells and restoring the sense of sight to patients suffering from retinitis pigmentosa [12].

Micro-channel tracks of noble metal coatings as electrodes, wires, and pads are among the most commonly used in the coplanar PDMS-based MEAs neural interfacing technologies [1,2,3,4,5,6,7,8,9,10,11,12,13,14,15], because the PDMS polymer provides easy fabrication of MEAs with high-density interconnects and integrated packing. For bioimplants application, thin films of platinum, gold, silver, as well as iridium oxides (IrO_x_), have been used for metal patterns, due to their high biocompatibility and corrosion resistivity. Various innovative approaches [1,2,3,4,5,6,7,8,9,10,11,12,13,14,15], which combined different methods and materials for preparing insulator substrate, its surface activation, and metal patterning, have been applied to produce high-resolution and high-density integrated PDMS-based stretchable MEAs devices for neural and muscular surface interfacing. Usually, the structure processes are complicated and multi-stepped, which include, for example, e-beam or thermal evaporation; electroplating of metal layers; spin coating and cured of the insulating PDMS layers; modification of PDMS surface from hydrophobic to hydrophilic by using oxygen plasma (shadow masks can be used for selective activation); lift-off method of photoresist by applying UV photolithography or ion etching for metal patterning the sample, etc. Depending on the manufacturing method used, other biocompatible metals such as Ti, Pd and/or Sn can be deposited or other organic (such as 2-hydroxyethyl methacrylate) materials to polymerize [2] and thus to enhance the PDMS adhesion to metal.

A significant simplification of producing channeled tracks for microelectrodes and microfluidic systems on polymers surface has been offered by applying the laser technique, especially in case of bioapplications.

Medical grade PDMS elastomers and other biocompatible polymers have been very successfully processed by nanosecond and femtosecond lasers, as the first step of fabrication of multielectrode arrays (MEAs) implantable neural devices and microfluidic systems [5,13,14,15,16,17,18,19,20,21,22,23]. Laser-induced local micro-structuring and chemical transformations lead to the formation of a highly active catalytic surface by increasing the surface energy and wettability of the polymers. The surface activation improves significantly the adhesion properties of the laser treated area, which facilitates the second step of the fabrication process, namely, the metal patterning. Metal deposition on the as-modified laser tracks can be conducted very successfully via electroless metallization, which is usually the most applied technique for the metallization of the polymers [24,25,26].

The main advantages of the laser-assisted fabrication due to which the polymer-based MEAs meet the high-technology demands for implantable neural devices are: (i) increasing the number of electrodes intercontacts by miniaturization of the laser-modified tracks; (ii) inducing effective surface activation and micro-structuring; (iii) contaminant-free processing; and (iv) providing further successful functionalization by deposition of high-quality metal coatings with well-defined microstructure and electrical characteristics.

In our previous investigations [27,28,29,30,31,32], we reported results on structuring and chemical activation of the surface of medical grade PDMS elastomers by UV, VIS, and NIR ns- and fs-laser processing conducted in air environment. Further, the laser modified PDMS surface has been successfully metalized via electroless plating of Ni [27,28] or Pt [32] on the laser tracks. The electroless metallization has been performed successfully without needing the application of the conventional procedure of preceding chemical cleaning and etching for micro-structuring and oxidation of the surface, followed by sensitization with Sn and chemical activation with Pd. Hydrazine hydrate is used as a reducer in the autocatalytic bath, the pH of the plating baths is >12 and the deposition temperature is set at 70 °C for Pt and 80 °C for Ni deposition. Depending on the hydrazine concentrations, some small differences in the crystallites structure have been observed, although the “spiky” origin remained. The aforementioned process of metallization is found to be much easier to apply than that one applied by Laude et al. [33] and Dupas-Bruzek et al. [34] after laser processing. In addition, selective metallization has been performed without applying masks or external templates. It is also worth noting our findings that the time interval between both processes—the laser treatment and the metallization—is not a critical parameter as it is proposed by Laude et al. [33] and Dupas-Bruzek et al. [34] in their experimental works on UV ns-laser surface modification of medical grade PDMS in air for miniaturization of nerve electrodes and tracks metalized by electroless plating of Pt. Numbers of references reporting on nanosecond or femtosecond laser processing by wavelengths in UV, ViS or NIR spectrum of different polymers (e.g., poly-methyl methacrylate (PMMA), polyethylene terephthalate (PET), polyimide (PI), polystyrene (PS), polycarbonate (PC), polydimethylsiloxane (PDMS)) are available in the literature [13,14,15,16,17,18,19,20,21,22,23,24,25,26,27,28,29,30,31,32,33,34,35]. Producing tracks (channels) and holes on polymer’s surface for the development the of micro/nanostructured based devices is of great interest for various applications in microfluidics, micromechanics, photonics, medicine, and biology. The high-quality of the polymers modifications has been evaluated in terms not only of their application purposes, but also in terms of laser control of their optical, wettability and/or structural properties as proposed D. Sola et al. [21,22,35] and other authors N.C.Y. Tham et al. [36], Bahador Farshchian et al. [37], and Carmela De Marco et al. [38]. This depends directly on the physical properties of the material, including optical absorption, thermal conductivity, density, hardness, etc., as well as on the laser beam parameters [13,23]. The ultrashort lasers have gained great attention in material processing due to the higher precision of local treatment in nanometer scale, lower heating effects and absence of re-deposition of debris (ejected material) beside the treated zones [18,28]. In the case of the ns-laser ablation of polymers in air the quality of the tracks and their contours tend to degrade and cannot ensure the high quality of the MEAs characteristics.

Concerning our experimental results, two main disadvantages of the ns-laser ablation of medical grade PDMS can be underlined: (i) the re-deposition of the ablated material (debris) in the zone adjacent to the ablated areas and (ii) the formation of very rough microstructures. However, our experimental findings, especially regarding UV ns-laser ablation, confirmed the results reported by other authors [26,39,40] that the ns-laser treatment of PDMS induced more prominent chemical transformations in the local area of impact rather than the fs-laser irradiation [21,22]. The significant chemical activation of the surface is the reason to prefer ns-laser treatment of PDMS elastomer. Therefore, the production of high-quality traces with well-defined and smooth contours and chemically activated surface by ns-laser irradiation of the biocompatible PDMS polymer without using masks or multiscanning ablation proves to be a great challenge. In this respect an advanced method of laser processing of medical grade PDMS in water surrounding was proposed by Stankova et al. in their 2018 patent application [41] and described in a chapter of the book [42].

Some References as Elaboudi I. et al. [43] report on the comparison between laser ablation efficiency in both air and water environments of polymers such as polymethyl methacrylate (PMMA), ethylene terephthalate (PET), polycarbonate (PC), (polyimide) PI, and polystyrene (PS) by excimer KrF laser. T.C. Chang et al. [44] have explained the dependence of etch depth on the laser energy fluence by existing polymer ablation models observed during UV (KrF) ablation of PMMA, polypropylene (PP), and polyethylene (PE) in air, methanol and ethyl alcohol. The effects of underwater ablation of PET with KrF laser on the surface morphology and chemistry has been reported by Jakub Siegel et al. [45]. The quality of channels obtained after ablation of PMMA in different environments (nitrogen, methanol, and water) with UV (248 nm) laser irradiation has been also investigated for the fabrication of microfluidic systems [46]. Black PMMA has been processed by NIR (1064 nm) wavelength in both ethanol and water for comparative studying the heat effected zone formation around the laser treated area [47]. However, the laser ablation of PMMA in organic liquid and especially in water resulted in obtaining channels with a wedge-shaped profile. Our preliminary experiments indicate that this type of profile shape could significantly hinder the effective deposition of metal during the autocatalytic metallization. To the best of our knowledge, so far, the production of channels by ns-laser ablation in water of optically transparent PDMS elastomer for MEAs neural implantable devices has not been reported on.

In this article, we report on simple approach for ns-laser ablation of optically transparent medical grade PDMS elastomer (without any UV additives) in a water environment by applying two UV lights (266 and 355 nm) of Nd:YAG laser system. To the best of our knowledge, by using this approach, for the first time we obtained free of debris tracks with regular contours and symmetric wide-opened profiles with a rounded (“U”) shape, which provide opportunities for subsequent qualitative functionalization by electroless metallization with platinum (Pt). The laser tracks possess a chemically activated and homogeneous surface microstructure, which ensures on one side the autocatalytic plating of Pt without preliminary sensitization by Sn and chemical activation by Pd, and on the other side facilitates the formation of platinum black layers with low sheet resistance—between 1 and 8 Ω/sq—in terms of their application as PDMS-based stretchable MEAs interfacing devices.

## 2. Materials and Methods

**Figure 1:**
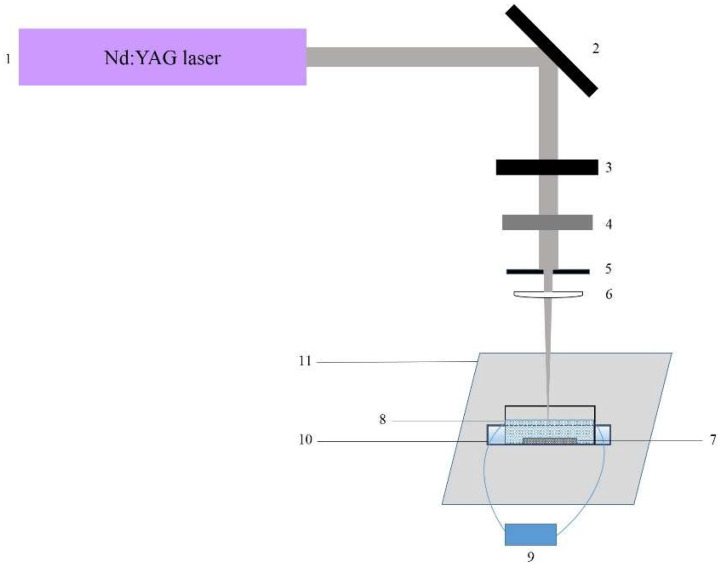
Experimental set-up of laser ablation of PDMS in water: (1)—Nd:YAG laser; (2)—mirror; (3)—attenuator; (4)—shutter; (5)—diaphragm; (6)—lens; (7)—PDMS sample; (8)—vessel with water; (9)—water pump; (10)—coolant tank; (11)—*x-y* translation stage.

Sheets of medical grade PDMS (MED 4860, NuSil, CA, USA) elastomer (with thickness between 80 and 200 µm), which is optically transparent in the UV-Vis-NIR spectrum, are processed by third (355 nm) and fourth (266 nm) harmonics of Q–switched Nd:YAG laser (LOTIS TII LS-2147, Minsk, Belarus) delivering 18 ns laser pulses at a repetition rate of 10 Hz and multimode laser beam. The samples are placed on the bottom of a glass vessel (with size—diameter of 100 mm and height of 15 mm) and immersed in double-distilled water with specific conductivity ~1.0 µS·cm^−1^. The water column above the sample surface is kept at a height of 8 mm. At this low height the possibility of the nonlinear absorption of laser beam by water is minimized. The water is set to circulate with a slow flow rate less than 5 mL·s^−1^ during the laser treatment, which provides dissipation of the ablated material. The water vessel is placed in another container (tank), which contains coolant with a temperature lower than the room temperature, see Figure 1. The aim is a faster evacuation of the heat delivered by laser irradiation from the ablation zone, and thus the formation of a heat-affected zone to be minimized. The polymer sample, the water container, and the coolant tank are fixed to a computer-controlled *x-y* axes translation stage. The laser beam is perpendicularly incident on the polymer surface and focused by a lens with a focal length of 100 mm. The focal spot diameter is defined experimentally after 20 pulse shots on the PDMS surface in a water environment for values of the laser energy, at which the efficient ablation starts for wavelengths of 266 nm and 355 nm, respectively. The laser ablation is performed by a single scanning of the samples in multi-pulse mode. The number of overlapping pulses *N* is defined by *N* = *a·f/s*, where *a* is the laser spot diameter, *f* is the pulse repetition rate and *s* is the moving speed of the stage. During all experiments the step of the *x-y* translating stage is fixed at 12.7 µm, which determines one-axis speed of 12.7 µm/s. Therefore, the number of overlapping pulses per unit area within the spot size is calculated to be 63 pulses for 266 nm and 72 pulses for 355 nm. The laser fluence is varied from 4.60 to ~50.00 J·cm^−2^ at the wavelength of 266 nm and from 7.70 to ~53.10 J·cm^−2^ at the wavelength of 355 nm. The laser beam parameters are listed in Table 1. The values of the laser fluences presented in the table are valid only for the first pulse hitting the polymer. As the laser beam scans the sample surface, the consecutive pulses overlap and cause changes to the PDMS optical properties by the incubation phenomenon. Due to this, defects are induced below the surface, which leads to significant increasing the optical absorption [29] and as a result effective ablation occurs. Continuous laser tracks with desired geometric dimensions (width, depth, length, and shape configuration) are successfully produced by scanning the samples at preliminarily established process parameters.

The PDMS samples are cleaned consecutively with ethanol and deionized water in an ultrasound bath and finally dried by an air stream, before the laser treatment. After laser treatment, the samples are metalized with noble metal Pt by using the electroless deposition technique (autocatalytic bath). The metallization is performed without usually preceding sensitization by Sn and chemical activation by Pd of the surface. The electroless plating process is based on a hydrazine hydrate reducer at an optimal concentration determined empirically. The bath for electroless deposition of Pt contains: K_2_Pt(NO_2_)_2_, NH_4_OH, NH_2_OH•HCl, Hydrazine hydrate (80% N_2_H_4_). The plating is performed at a temperature of 45 °C and pH 11.4. In this case, during the electroless deposition of Pt evolution of nitrogen gas is possible.

The characterization of the laser processed areas is performed by different analytical techniques including: optical microscopy (Zeiss Opton) for observing the visible permanent surface modifications; VK-9700K Color 3D Laser Microscope (KEYENCE) for viewing the laser trucks’ depth and profile; scanning electron microscopy (SEM) with FEG SEM Hitachi SU-70 and SEM/FIB (Lyra/Tescan dual beam system, Brno, Czech Republic) for detailed surface morphological imaging of the laser-treated surface and the Pt coatings structures. µ-Raman spectroscopy (RMS-310 µ-Raman spectrometer (Photon Design, Tokyo, Japan) equipped with a laser operating at a wavelength of 532 nm), and X-ray photoelectron spectroscopy (XPS) measurements are performed in a PHI model 5600 system equipped with an Omni Focus Les III (ULVAC-PHI, Chigasaki, Kanagawa, 253-8522, Japan) using a standard Mg Kα X-ray source at a high voltage of 15 kV, 300 W. The PHI Multipak 9 software is used for data treatment and interpretation. The calibration is done by normalizing the C 1 s line of adventitious adsorbed hydrocarbons on silver folio to 285.0 eV) for studying the chemical composition and surface chemical transformations.

## 3. Results and Discussion

### 3.1. Laser Processing

The microchannels on the PDMS surface are fabricated by multipulsed laser ablation in water after single scanning the surface (for each track). The numbers of overlapping pulses (N) for both UV lights are set to be as high as possible allowed by the equipment used. They are limited by the laser spot on the surface, the repetition rate of the laser pulses (10 Hz), and the stage speed, all of which are fixed. The laser spot size is determined mainly by the lens focus distance (100 mm) and the wavelength. The minimum moving speed of the stage (12.7 µm/s) is set to ensure precise scanning of the surface. Thus, the energy delivered in a unit area during laser ablation can be controlled by the energy per pulse. Tracks with continuous and uniform ablation depth along their length are obtained at laser fluences higher than 10.40 J·cm^−2^ for 266 nm and at fluences higher than 14.05 J·cm^−2^ for 355 nm. These values are higher than the ablation threshold for both wavelengths at multipulsed mode and considered as fluences, at which efficient ablation along the track initiates. From Table 1, it is evident that higher values of the laser fluence for the wavelength of 355 nm are needed in comparison with irradiation at 266 nm to start laser ablation of PDMS and to cause similar etch depth. These results are consistent with those obtained in our previous investigation of the optical properties of PDMS during ns-laser treatment in air [29]. This dependence can be explained on one side by the lower optical absorption of the material (nearly two times less) at 355 nm (linear absorption coefficient is about 7.38 cm^−1^, the penetration depth of 1354 nm) concerning this at 266 nm (linear absorption coefficient is about of 14.9 cm^−1^, penetration depth of 669 nm), and on the other side by the lower photon energy of the laser irradiation at 355 nm (3.5 eV). The absorption coefficient and the penetration depth are calculated following the Beer–Lambert law for an average thickness of the sample of 170 µm, as the scattering losses are ignored. It is worth noting that the absorption and refraction of the laser beam by water can be ignored, since the water absorption at 266 and 355 nm is very low (the absorption coefficient is of the order between 10^−3^ and 10^−4^ cm^−1^), the laser beam is normally incident to the water surface, and the water column height is low enough (8 mm) to induce nonlinear effects or significant changes of the focused spot size [48] on the sample surface.

According to the investigations reported in the literature [49,50,51,52,53,54], complex thermodynamic and acousto-mechanical processes take place during laser ablation in water and related effects can be observed: plume expansion, laser-induced cavitation bubbles, bubble collapse, shock waves, etc. In the case of water flow, waves and turbulence can arise and disturb the laser beam propagation. However, studying such processes during laser ablation of PDMS in water is not subject to the current work. During the experiments presented in Section 2, we observed: (i) the formation of visible bubbles and acoustic effects; or (ii) the formation of waves on the air-water interface and water splashes, and the related laser beam propagation perturbance are found to occur when the ablation takes place: (i) simultaneously in still water, high pulse energy delivering (F_266 & 355 nm_ > 10 J·cm^−2^) (at minimum stage moving speed of 12 µm/s and a high number of overlapping pulses—63 or 72), or at (ii) water column higher than 8 mm in a narrow vessel with a diameter lower than 50 mm regardless the pulse energy.

To explain the reasons for this phenomenon additional characterizations and diagnostic techniques should be applied, which will to be the topic of future work.

**Figure 2:**
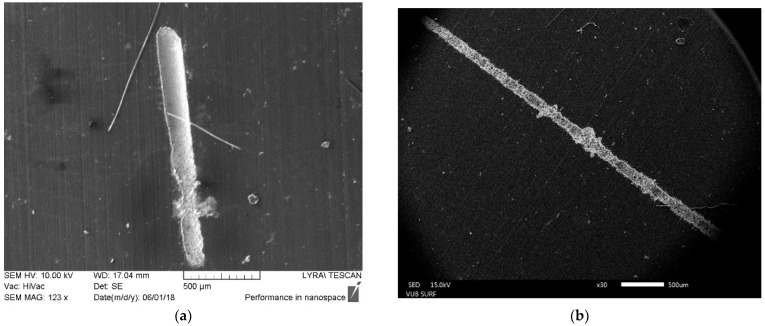
SEM images of tracks with worsening morphologic quality obtained on PDMS surface by laser ablation in water with wavelengths of: (**a**) 266 nm and 28.07 J·cm^−2^ and (**b**) 355 nm and 30.83 J·cm^−2^ when bubbles are induced in the zone of the laser beam spot. The tracks are metalized with Pt.

The presence of bubbles in the zone of laser beam spot during ablation in water is extremely undesirable in our experiments, since they always lead to worsening morphologic quality of the laser-treated surface such as irregular counters, debris and heat-affected zone HAZ could be observed beside the tracks, as it is seen in Figure 2. This is out of the strict demands for producing high-quality neural interface devices, which is our final target.

In this respect, to minimize or even to avoid the effects listed above the output energy per pulse is reduced nearly 8 times by an attenuator and a diaphragm both placed in the beam propagation path (before the lens). A water pump is mounted to keep slow flow rate between 3 and 5 mL·s^−1^. According to the results obtained, this flow rate is high enough for decreasing the plasma shielding effect (laser beam absorption by the plasma plume) and removing debris far from the ablation zone. Empirically, it has been established at this range of rate values the flow prevents the formation of bubbles during the ablation. Moreover, cooling the water additionally by coolant keeps its temperature lower enough than the room temperature. We believe that cool water contributes to (i) increase the convective heat transfer near the zone of ablation due to the temperature gradient induced by both the coolant and the water flow, and to (ii) enhance the dissipation of the ablated material and move it away from the ablation zone in addition to the water flow effect. Also, flow rate and cooling of the water, reduce the formation of HAZ near the laser-treated zone, proven by optical microscopy images in Section 3.2.

If the flow rate increases higher than 5 mL/s, the formation of bubbles in the ablation zone and waves at the air-water interface are also observed. Moreover, bubbles are observed to form more often at irradiation with a wavelength of 355 nm rather than 266 nm. This could be due to the higher thermal effect induced when higher energy is delivered through the water during irradiation with 355 nm compared to the irradiation with 266 nm, as is commented above and in detail in our previous work [29].

Additionally, the water volume and the vessel dimensions are found to be crucial for the formation of waves on the air-water interface and water splashes during the laser ablation. The water column height between 8 and 10 mm poured in wide (diameter of about 10 mm) and shallow vessel (with height up to 20 mm) is empirically established as optimal to avoid the indicated effects during ablation with laser fluences higher than 10 J·cm^−2^ at both wavelengths.

**Figure 3:**
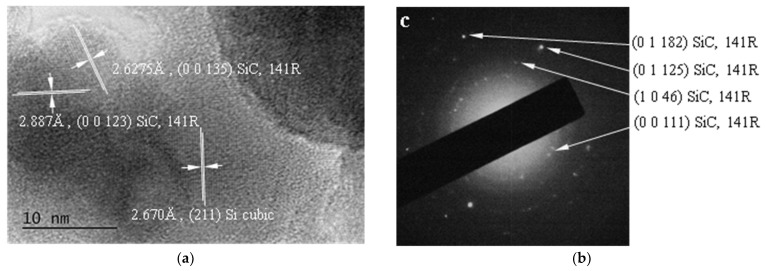
HRTEM (**a**) and SAED (**b**) images of the plasma plume products deposited on copper grids during the ns-laser ablation of PDMS in air with 266 nm and fluence ~4 J·cm^−2^ reveal presence of polycrystalline SiC and crystal Si phases.

Besides the laser-induced cavitation bubbles, other causes that could contribute to bubble formation should be considered. The chemical structure of PDMS consists of repeating monomer [SiO(CH_3_)_2_] units with the main chain of Si-O-Si and CH_3_ radicals bonded to the Si. Therefore, during the laser ablation of PDMS elastomer an evolution of C-radicals, CO_2,_ and/or molecular oxygen (O_2_) is possible [39]. The formation of inorganic products such as crystal silicon (c-Si) and crystalline and amorphous phases of carbon, confirmed by µ-Raman spectroscopic and XPS measurements in zones of the laser-treated area, supports this assumption. In addition, by using transmission electron microscopy (TEM), the images are presented in Figure 3, an investigation of the plasma plume products deposited on copper grids during the ns-laser ablation of PDMS in air with 266 nm and 355 nm reveals the presence of polycrystalline SiC and crystal Si phases. The carbon phases are not revealed since the grids are carbon-supported. The presence of inorganic products in the plasma plume during the ablation of PDMS proves its chemical decomposition, which is related to releasing the gaseous phases, as mentioned above. Also, the evaporation of water in the near field of the laser beam could occur, because of the increasing local temperature due to the highly intensive UV laser irradiation and following ablation dynamics effects. This is possible to induce the decomposition of water. The gaseous species can be released and expelled along with the other particles in the plasma plume and thus give their contribution to the formation of the bubbles. However, our observation is that once the bubbles are formed above the sample surface it expands and the quality of the laser track drops dramatically. 

Our preliminary experimental investigation credibly contributes to the establishment of the optimal process parameters shown in Table 1 and described in Section 2, at which the hydrodynamic and plasma dynamics effects, described above, as well as the re-deposition of the ablated material beside the tracks and HAZ formation during the ns-laser ablation of PDMS polymer in water can be decreased or avoided. The adjustment of all processing parameters (laser fluences between 10.40 and 49.96 J·cm^−2^ for 266 nm and between 14.05 and 44.53 J·cm^−2^ for 355 nm, high number of pulses, water flow rate < 5 mL/s and column height of 8 mm) allow the production of high-quality laser tracks with tunable size and well-defined open “Ü”- shaped profile in accordance with the application requirements for achieving high-definition tracks free of debris. It is important to emphasize that the tracks obtained by ablation with 266 nm revealed more uniform and homogeneous microstructure along the length possibly due to the higher optical absorption yielding more efficient ablation in comparison to the laser irradiation generated at a wavelength of 355 nm.

### 3.2. Morphological Structure

**Figure 4:**
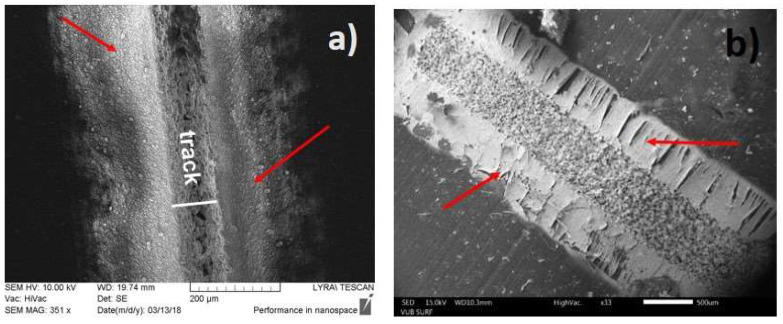
(**a**) Laser track after ns-laser ablation of PDMS sample in air with a wavelength of 266 nm, the beam spot is in the focal plane, the red arrows show the debris beside the track; (**b**) Pt (platinum) coating deposited by electroless metallization of a track obtained by ns-laser ablation of PDMS sample in air with 266 nm wavelength, the beam spot is out of the focal plane, the red arrows show the debris beside the track, on which enough quantity of Pt is deposited.

In our previous investigations reported in Refs. [27,29] the direct ns-laser writing on PDMS MED 4860 surface in air environment by using wavelengths 266, 355, 532, and 1064 nm have always revealed one drawback—re-deposition of the ablated material (debris), which has also been reported in ref. [18,20]. During multi-pulse laser ablation, the ejected material is deposited on the pristine surface adjacent near to the laser-treated area as is shown in Figure 4a. The debris are highly undesirable since incorporate a negative effect on the process of subsequent functionalization of the as-modified surface by electroless Pt metallization. Most of the Pt ions contained in the metallization bath are deposited predominantly on the debris ejected beside the tracks, see Figure 4b. This leads to a rapid depletion of the Pt ions contained in the plating bath and wasting an expensive noble metal such as platinum is. This, in turn, is a precondition for several undesirable effects, which can worsen the microelectrodes arrays’ quality and inability to miniaturize the chip (namely, inability to increase the number of the tracks) as well as a short circuit between the metalized tracks. Therefore, to meet the requirements for precise and high definition MEAs devices for neural implantable interfacing technologies, is necessary to obtain high-quality laser modification by excluding re-deposition of debris and thus to increase the number of tracks (electrodes) per unit area, i.e., miniaturization of the chip. Production of free of debris laser-processed tracks permit their further successful functionalization by Pt coatings.

The other observed disadvantage of the ns-laser ablation of this polymer in air environment is the formation of the wedge-shaped profile of the channels, which are obtained after ablating in the focal plane of the lens (see Figure 4a) for obtaining the small-sized track. Also, the formation of rough and inhomogeneous relief with deep pores inside the tracks interferes with further deposition of a continuous metal layer, and as a result the electric circuit could not be qualitative and reliable for the intended application as MEAs.

To overcome these shortcomings, we successfully provide two different approaches of ns-laser processing of PDMS elastomer substrates. The first one is laser processing on the polymer surface by laser ablation in water [41,42]. The second one is the ablation in air of the PDMS surface, which preliminary is pre-coated with a thin layer of PMMA by spraying. Here, after performing ablation in air this thin film of PMMA is removed by cleaning in an ultrasonic bath. 

**Figure 5:**
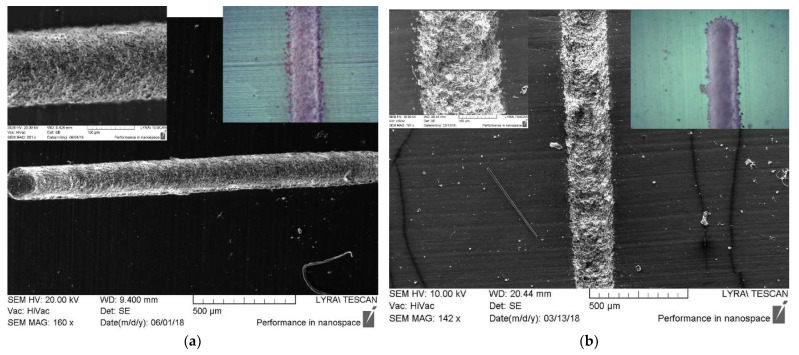
SEM images in different magnifications are applied in order to demonstrate production of free of debris laser tracks after ns-laser ablation of PDMS polymer in water. No evidence of deposition of ejected material and heat-affected zone is observed near the place of ablation. The insets present higher magnification of the SEM images and images taken by optical microscope: (**a**) 266 nm and fluences higher than 28.07 J·cm^−2^; (**b**) 355 nm and fluences higher than 21.75 J·cm^−2^.

**Figure 6:**
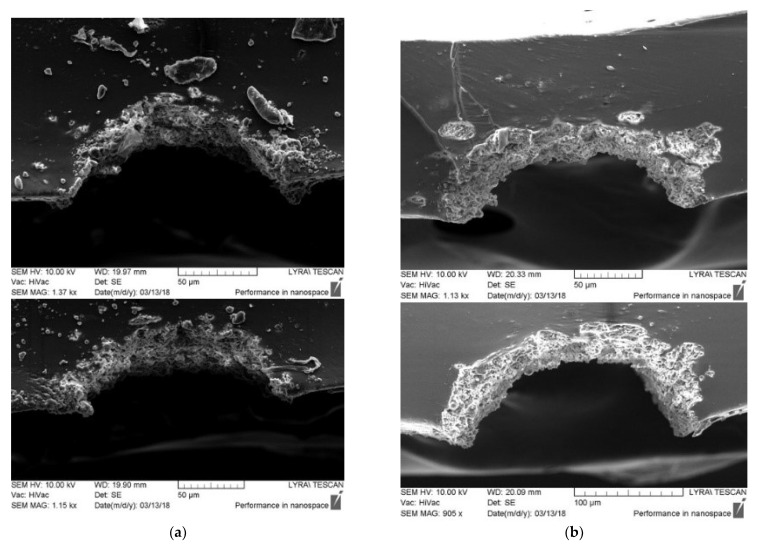
SEM cross section images of the laser tracks produced after ns-laser ablation of PDMS polymer in water present the formation of Profiles with regular contours, and symmetric and wide-opened U-shape, with: (**a**) 266 nm and fluences higher than 28.07 J·cm^−2^; (**b**) 355 nm and fluences higher than 21.75 J·cm^−2^.

During the one-scanned multi-pulsed laser ablation in water tracks with homogeneous depth and well-defined borders are obtained at fluences higher than 10.40 J·cm^−2^ and 14.05 J·cm^−2^ at wavelengths of 266 nm and 366 nm, respectively. No evidence of deposition of ejected material and heat-affected zone is observed near the place of ablation. The produced tracks are free of debris with uniform nanostructured relief (see in Figure 5). The profiles with regular contours and wide-opened symmetric U-shape are formed as a result, (Figure 6). The ablation at lower fluences is not efficient and the laser tracks obtained are not continuous along their longitudinal direction and also lose their linear contours.

Highlighting, the tracks obtained by ns-laser processing of PDMS elastomer in water, especially with a wavelength of 266 nm and fluences higher than 10.40 J·cm^−2^, are of very high quality, perfectly comparable with the quality of these produced by fs-laser processing of PDMS samples in air environment, reported previously in our works [27,28,31] as well as by F. Baset et al. [18]. Briefly, the ns-laser processing of PDMS elastomer in water causes the formation of a similar nanostructured surface with uniform relief topography, wide-open tracks with regular borders, as is evident from the electron and the optical microscopes’ images, Figure 5. A comparison with the results obtained after laser processing of polymers (PMMA, PDMS, PGS, APS) and already reported by other authors [13,17,38] revealed that we have succeeded to produce these high-quality laser tracks by applying only one-scanning ablation with multimode nanosecond laser irradiation at a low repetition rate. Namely, for such parameters, the nanosecond lasers are avoided using for micromachining, because of the lower quality (in terms of the characteristics described above) of the laser tracks compared to the femtosecond lasers abilities.

The size of channels becomes smoothly wider and deeper at both wavelengths applied by increasing the energy, as is expected. However, it is observed that the profile width increases faster than the profile depth with increasing laser fluence for both wavelengths applied. This could be due to hydrodynamic effects related to the plume expansion and water confinement during the ablation, and to the applied laser energy, all in a strong correlation to the material properties. The channels’ width and depth can be easily controlled considering their strong dependence on the laser fluence. Corresponding values are shown in Table 1.

Attention must be drawn to the comparison of the channels’ profiles produced by laser ablation in a different environment—air and water—showing two intriguing peculiarities: (1) by increasing laser fluence the width and depth also increase, valid for both environments; (2) in air deep V-shaped profiles are formed like in ref. [17], which reports the same profile shape after single-scanning fs-laser ablation of polymers (PMMA, PS) with a wavelength in NIR spectrum—800 nm and formation of highly open U-shaped profiles after multiscanning ablation. While in the case of single-scanning nanosecond laser ablation in water the profiles are wide open with a symmetrical U-shape. To summarize, the water contributes to the high-quality performance of the laser tracks simultaneously by carrying the debris away and promoting the formation of desirable profiles shape, in terms of the intended MEAs application. An explanation of the difference between profiles obtained after ablation in air and water could be given after applying characterizations and diagnostic techniques, which are not the subject of this topic. However, we assume that the prominent difference is due to the different physics effects that occur during ns-laser ablation in air and water. As in the case of ablation in water environment, correspondingly related processes can increase the laser ablation efficiency. In water environment additionally to the impacts of plasma expansion dynamics, intense water jet-induced impulses can be produced due to the cavitation bubble collapse in the vicinity of the polymer surface [55]. Also, the water confinement effect can increase the backpressure (recoil) effect on the laser-treated zone during plasma expansion [43,49,54]. Further, modulations in quantum mechanical aspect (such as decreasing of the phase velocity of the photons and increase of their momentum can be responsible [56], and geometric distribution transformations [48] of the laser beam could be expected during its propagation in the dispersive media with a refractive index higher than 1 such as the water is, despite the low height of the water column.

**Figure 7:**
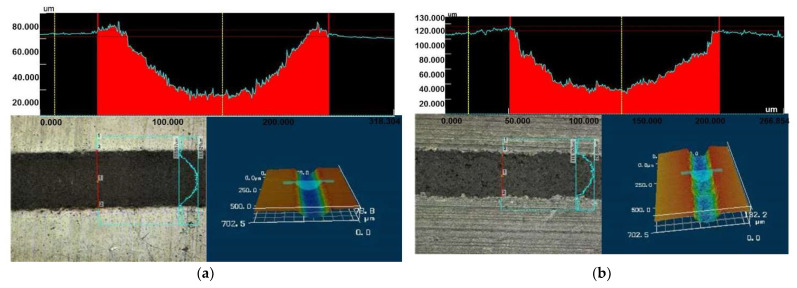
3D Laser microscope views of the tracks produced by: (**a**) 266 nm laser pulses with fluence 42.66 J·cm^−2^; and (**b**) 355 nm laser pulses with fluence 37.68 J·cm^−2^. It can be seen no debris are re-deposited after laser ablation near the tracks. HAZ also is not formed.

The 3D color laser microscope analysis confirms the results obtained by scanning electron microscopy. Examples of tranches, produced by 266 nm and laser fluence of 49.96 J·cm^−2^, and the tranches, produced by 355 nm and laser fluence of 37.68 J·cm^−2^, are presented in Figure 7a,b, respectively. As it can be seen, clear and well-defined tranches with homogeneous surface microstructure are produced in both cases. The profiles’ shape seems perfectly symmetrical. Moreover, no debris redeposited and not affected zone adjacent near to the tranches are observed. This picture is typical for the tracks produced at fluences higher than 10.40 J·cm^−2^ and 14.05 J·cm^−2^ at wavelengths of 266 nm and 366 nm, respectively, especially at 266 nm.

By controlling the translation of the x-y stage different configurations of tracks can be obtained. It allows the complex design of microelectrode arrays relevant to the specific technology requirements. Particular structures with specific size and shape characteristics (depth, width, profile shape) could be obtained under controlling laser fluence, moving stage speed (respectively, the number of overlapping pulses) as well as the water parameters (column height, flow rate, temperature, water purity). According to our experimental findings, the optimal laser fluences, which provide conditions for high quality and permanent stability of the ns-laser ablation performance are fluences higher than 10.40 J·cm^−2^ for 266 nm and 14.05 J·cm^−2^ for 355 nm, especially at 266 nm for higher optical absorption of the PDMS elastomer at this wavelength.

It is important to underline that treating the sample in water with a specific electrical conductivity less than or equal to 1.0 µS·cm^−1^, the resulting structure is applicable for medical purposes.

### 3.3. Chemical Composition

**Figure 8:**
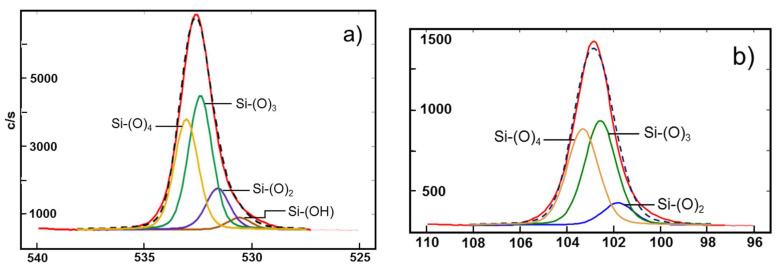

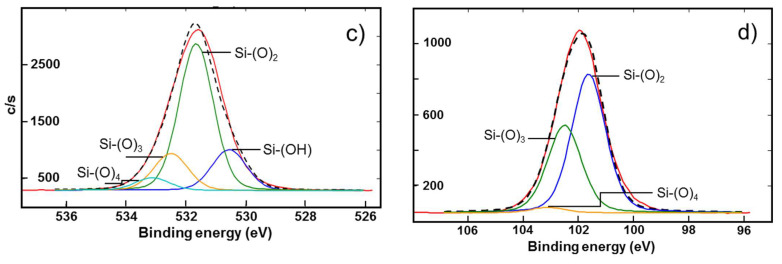
Curves fitting of XPS spectra of O1 s (**a**,**c**) and Si 2p (**b**,**d**). Laser ablation of PDMS with 266 nm: (**a**,**b**) in air with fluence 4.3 J·cm^−2^; (**c**,**d**) in water with fluence 28.07 J·cm^−2^. Dashed line is the fitting curve.

XPS study of the surface structure of PDMS reveals that chemical degradation occurred after UV laser treatment in water. The results of Si(–О)n components in Si 2p and O 1 s peaks are presented in Table 2 after their deconvolution. The binding energy (BE) value at 101.63 eV is assigned to a pristine organic silicone phase or PDMS backbone Si(–O)2. While the peaks Si(–O)3 at 102.6 eV and Si(-O)4 at 103.3 eV measured in the laser-treated area correspond to the inorganic silica-like (SiOx) phase. The portions ratio between pristine and processed surface could be presented as approx. 60%:40%. The high portion of inorganic silicon indicates that the PDMS surface suffered significant chemical changes after laser exposure as it was observed to happen after laser ablation in air. The deconvolution of the O 1s peak reveals a hydroxyl group (–OH), which is attached to the organic molecule by the appearance of a peak at 532.55 eV. This binding energy value can be assigned to the formation of a Si(–OH) functional group, which confers the hydrophilic properties of the laser-treated surface. The portion of Si(–OH) is 17.3% when processing is performed in water (see Table 2) with respect to 4% when processing is in air (established in previous our works [30,31]). Increasing the Si(–OH) component after laser ablation in water is considered as one of the main factors providing to a great extent of the hydrophilicity and the highly efficient deposition of Pt only on the processed area via electroless metallization—which is one wet process taking place in a complex chemical solution. The results presented in Table 2 correlate to curves of O 1s and Si 2p peaks, presented in Figure 8.

**Figure 9:**
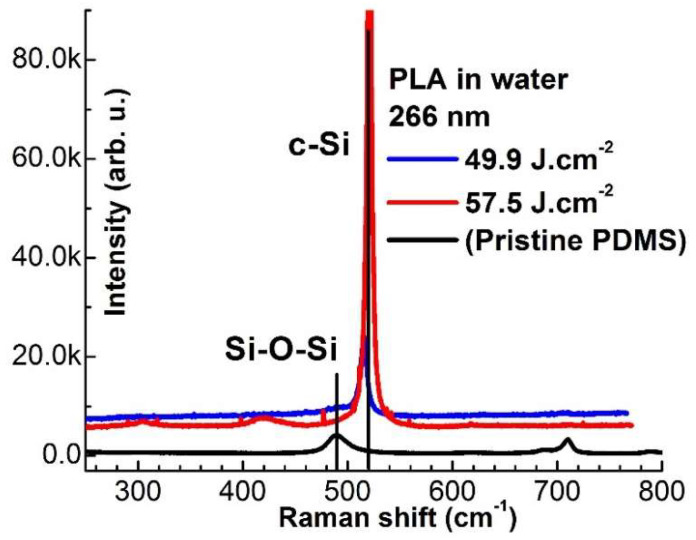
Raman spectra of PDMS polymer processed by 266 nm laser pulses in a water environment. Similar Raman spectra are obtained after processing with 366 nm. The Raman spectra demonstrate chemical activation of the surface after UV laser treatment with all fluences applied.

These results are convincingly confirmed by measurements with μ-Raman spectroscopy of the samples processed by UV laser in water. A strong and sharp peak appeared at 516 cm^−1^ and shifted to 520 cm^−1^ with increasing the laser fluence for the case of both wavelength (266 and 355 nm) applied. The peak is ascribed to mono and/or polycrystalline silicon (c-Si). Also, it is observed that its intensity raises with laser fluence, whereas the intensity of the peak at 489 cm^−1^, corresponding to the O-Si-O, sharply dropped, see Figure 9. The same tendency is observed for PDMS samples irradiated in air with ns-laser wavelengths in the UV-ViS-NIR spectrum range.

To emphasize, both analyses confirmed that UV ns-laser processing of PDMS polymer in water causes chemical transformations and increases its chemical activation and hydrophilic properties. 

### 3.4. Electroless Metallization with Pt

**Figure 10:**
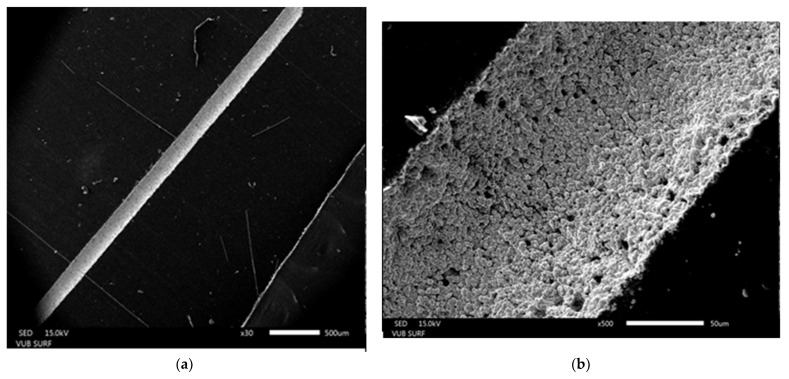

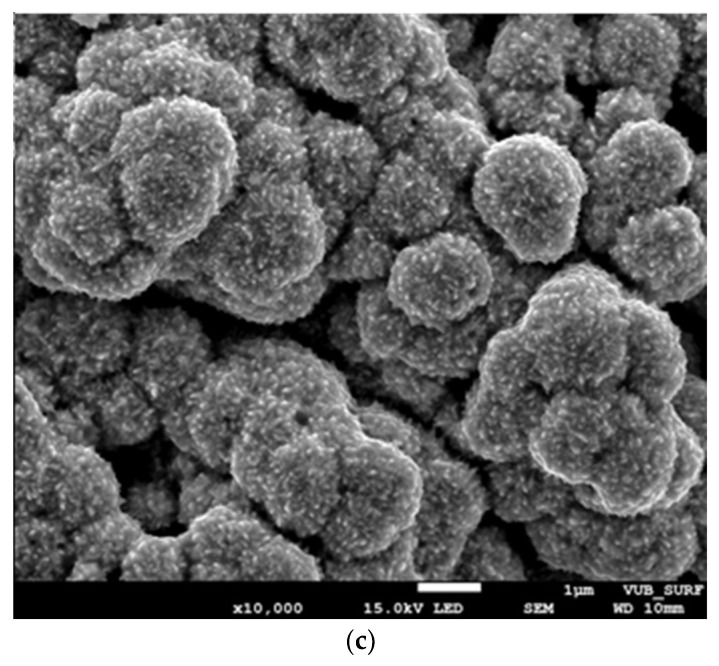
SEM images of electroless deposition of Pt layer on the track obtained by ns-laser ablation of PDMS (wavelength of 266 nm, fluence 35.37 J·cm^−2^) in water. High-quality metal coating only on the laser-treated area is obtained. Different magnifications are applied to demonstrate (**a**,**b**) the absence of debris near beside the laser tracks, and (**c**) the cauliflower-like structure of the Pt layer, consisting fine nanograins.

Thus, the chemically activated surface with increased hydrophilicity and homogeneous microstructure in the ablated zone and the absence of debris near ensure a very efficient process of deposition of Pt only within the laser ablated area by electroless metallization. SEM examinations show that platinum completely covers only the laser-treated areas (Figure 10). Circular grains are observed, which gather in adjacent wrists to form a cauliflower-like structure, which is typical for the electroless deposited coatings. Each grain resembles a flower with very fine spikes and petals with a size up to 100 nm. Thus, platinum black coatings consisted of very small particles in size, which yields very high surface area. In addition, the optical microscope images reveal that the surface of the metal coatings appears as uniform black layers. This proves that the tracks are covered by platinum black films (their surface is rough and opaque and seems black relative to the transparent polymer) and do not have a silvery shiny look like a platinum film without having grain’s structure. This is the most desired performance of platinum electrodes in MEAs development because of their effective conducting characteristics [5,12,57]. Platinum-black microelectrodes have been preferable used in producing implantable neural and neuroprosthetics interface devices performed like neural networks [1,5,12,57]. The sheet resistance of the Pt coatings deposited on our laser tracks is measured to be very low, varying between 1 and 8 Ω/sq. These results are much promising and prove that the advanced approach for ns-laser ablation of PDMS in water activates the polymer surface most appropriately for successful further functionalization by electroless metallization.

**Figure 11:**
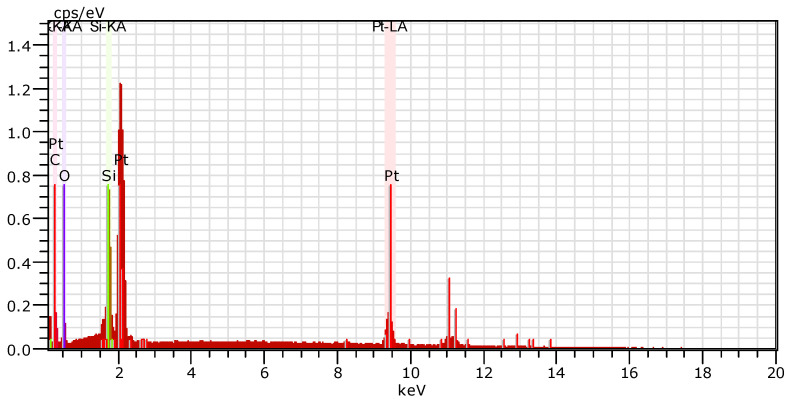
EDX image presented an elemental spectrum of electroless deposited Pt layer on the track obtained by ns-laser ablation of PDMS (wavelength of 266 nm, fluence 35.37 J·cm^−2^) in water, confirming that the metal crystals are truly platinum.

In Figure 11 EDX analysis of the deposited Pt layer is presented. This determines the crystals formed during the electroless metallization are truly platinum.

The deposition of such a layer would significantly improve the electrical characteristics and the efficiency of the potential device because of its use as a neural implant. As a result of this treatment, we observed that the consumption of the deposited metal (Pt) significantly decreases and, accordingly, no rapid depletion of metal in the autocatalytic bath happens.

Based on our preliminary and also on the current results, can be summarized that two main factors facilitated the electroless metal deposition and hence favorably influenced the formation of fine Pt grains: the first is the nanostructured morphology of the surface and the second is the hydrophilicity within the processed area both induced during ablation in water by UV ns-laser irradiation.

## 4. Conclusions

To the best of our knowledge, we report on results related to a novel advanced method for micro/nanostructuring and chemical activating polymer surface, in the particular medical grade of PDMS elastomer, by single-scanned laser ablation in water by applying a certain number of overlapping nanosecond UV (266 nm and 355 nm) laser pulses on a unit area. Circulating water with a low rate and column height are fixed, while the laser fluence is the variable parameter. To highlight, the results obtained are: (1) free of debris laser tracks with (2) symmetric wide-open and U-shaped profiles with regular and smooth edges and (3) uniform nanostructured topography is produced; (4) no adjacent heat-affected zones are observed; (5) hydrophilic properties of the laser-treated PDMS surface are induced by forming Si(-OH) functional group, which confer hydrophilic properties of the laser treated surface. The properties from (1) to (5) provide further (6) highly efficient functionalization of the laser-treated surface by autocatalytic (electroless) deposition of platinum (Pt) excluding preliminary sensitization with Sn and chemical pre-activation with Pd (these two steps commonly precede the autocatalytic plating); (7) formation of platinum black layers with a cauliflower-like structure consisting fine nanograins with (8) low values of sheet resistance between 1 and 8 Ω/sq; (9) the adjacent zones near the track contours are clear and not metalized.

The as-obtained metalized structures can meet the requirements in diverse application areas of stretchable MEAs interfacing technologies, but mainly in engineering solutions for the fabrication of high-definition flexible in-vitro and in-vivo neural networks. The proposed method could significantly contribute to: (1) miniaturization of the MEAs; (2) increasing the sensitivity and selectivity to the recorded and/or stimulated nerve pulses; (3) high mechanical matching between the device and the soft tissues; (4) low-cost and free of chemical contaminants process, since it is a no clean room-required method of fabrication.

## 5. Patents

The current results have been previously reported as a Patent application under number No 112728/03.05.2018 in the national patent office of Republic of Bulgaria. At present, this is a patent granted by the National Patent Office of the Republic of Bulgaria with registration number BG 67340/03.06.2021.

## Figures and Tables

**Table 1:** Summary of the laser beam parameters and the size of the tracks after laser treatment of the PDMS surface.

266 nm Laser Fluence (J·cm^−2^)	266 nm Track Profile (μm)		355 nm Track Profile (μm)		355 nm Laser Fluence (J·cm^−2^)
	Width	Depth	Width	Depth	
4.64	-	-	-	-	7.71
6.90	-	-	-	-	10.62
10.40	90	27	68	25	14.05
12.82	109	53	112	33	17.30
17.24	128	53	106	64	21.75
22.77	136	51	129	67	27.06
28.07	144	52	139	82	30.83
35.37	160	55	141	98	37.68
42.66	183	48	165	78	44.53
49.96	207	48	185	90	53.10

**Table 2:** Comparison of XPS data of the PDMS surface after ns-laser ablation within water and air.

Ns-Laser Ablation		Silicon Chemical Environments and the Corresponding Si 2p BE			Oxygen Chemical Environments and the Corresponding O 1s BE.			
**In water**	**Chemical Structure**	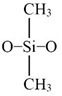	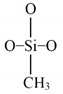	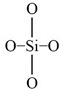	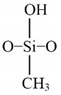	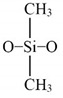	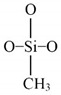	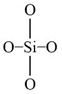
	**Abbreviation**	Si(–O)2	Si(–O)3	Si(–O)4	Si(–OH)	Si(–O)2	Si(–O)3	Si(–O)4
	**Experimental BE (eV)**	101.63	102.60	103.30	530.55	531.65	532.49	533.11
	**Reference BE (eV)**	101.79	102.67	103.30	530.60	532.00		533.20
**In water**	**Calculated from fitting in Figure 8 (%)**	59.80	37.80	2.40	17.30	62.00	15.50	5.20
**In air**	**Calculated from fitting in Figure 8 (%)**	25.06	12.80	62.14	4.00	32.16	21.78	41.66
